# Human sporotrichosis in the Amazon: an emerging zoonosis and its clinical-epidemiological challenges

**DOI:** 10.1590/0037-8682-0560-2025

**Published:** 2026-09-14

**Authors:** Ana Carolina Magalhães Nascimento, Luiza Rennó Rocha De Oliveira, Maria Amélia Lopes dos Santos, Elcilane Gomes Silva, Francisca Regina Oliveira Carneiro, Renata Mie Oyama Okajima, Carla Andrea Avelar Pires

**Affiliations:** 1 Universidade do Estado do Pará, Departamento de Dermatologia, Serviço de Referência Especializado em Dermatologia, Belém, PA, Brasil.

**Keywords:** Sporotrichosis, Epidemiology, Zoonosis, Itraconazole

## Abstract

**Background:**

The number of sporotrichosis cases in Brazil has been continuously increasing, primarily due to zoonotic transmission in urban areas. This study aimed to analyze the occurrence of human sporotrichosis in a reference center in the Amazon region.

**Methods:**

This case series study analyzed the medical records of patients diagnosed with sporotrichosis at a dermatology reference service in Northern Brazil.

**Results:**

Most patients presented with the localized cutaneous form. Diagnosis was confirmed using clinical and epidemiological criteria, histopathology, and/or culture.

**Conclusions:**

Diagnostic suspicion, especially in endemic areas, and early treatment are essential to reduce morbidity, sequelae, and adverse outcomes.

Sporotrichosis is a chronic granulomatous infection caused by thermodimorphic fungi of the genus *Sporothrix* ^1^. The disease has a global distribution and is considered endemic in several Latin American countries, including Brazil, Colombia, and Argentina^2^ ^,^ ^3^. 

In certain regions of Brazil, sporotrichosis is popularly known as the “cat disease” owing to its zoonotic transmission through felines. Although it was historically described as a saprophytic disease transmitted through inoculation of fungal propagules present in thorns, soil, or organic matter, the zoonotic route has emerged as an important mode of human transmission, resulting in a substantial increase in cases and rapid dissemination in urban areas. This scenario has established sporotrichosis as a major public health concern^4^.

Given the expansion of zoonotic transmission and the growing number of cases, human sporotrichosis was included in the National List of Compulsory Notification through Ordinance GM/MS No. 6,734, issued on March 18, 2025 (Portaria GM/MS nº 6.734, dated March 18, 2025). This regulation formalizes the mandatory registration of cases in the SINAN system, thereby supporting epidemiological surveillance and control actions^5^.

Clinically, sporotrichosis manifests as lymphocutaneous, localized cutaneous, and disseminated forms, while extracutaneous forms occur mainly in immunocompromised patients^6^ ^,^ ^7^. Although sporotrichosis is generally associated with low morbidity, except in disseminated cases, it may have a profound impact on patients' functional status and psychosocial well-being, substantially impairing their quality of life^8^.

Treatment should be individualized, with itraconazole as the drug of choice, and the dosage should be adjusted according to disease severity. Potassium iodide and terbinafine may be used in specific situations, whereas amphotericin B is reserved for systemic or severe cases. Treatment is prolonged, and adherence directly influences treatment outcomes, including disease resolution and the prevention of disease recurrence^9^.

Therefore, the analysis of epidemiological data, clinical characteristics, and therapeutic outcomes in patients with sporotrichosis is essential for understanding disease patterns and progression. This evaluation is particularly timely following the inclusion of sporotrichosis in the National List of Compulsory Notification, which provides data to support integrated health strategies aimed at disease control.

This was an observational, descriptive, retrospective, single-center case series study conducted at a reference center for the provision of secondary dermatological and tropical disease care in Northern Brazil. This study was approved by the University Research Ethics Committee (Opinion No. 5.647.696/2022) and authorized by the Dermatology Service Coordination. This study followed the CAse REport (CARE) guidelines for case reports and case series.

Convenience sampling was employed, consisting of patients of both sexes and all age groups who were treated at the service between June 2020 and June 2025 and had a confirmed diagnosis of sporotrichosis based on clinical, histopathological, and/or mycological culture criteria. Data were collected using a standardized protocol developed by the authors and applied to the patients’ medical records. Data not available in the medical records were supplemented through telephone contact with patients or their guardians. 

The findings of this study highlight the epidemiological transition of sporotrichosis from an occupational sapronosis to an emerging urban zoonosis, driven by the expansion of *Sporothrix brasiliensis* in Brazil^9^ ^,^ ^10^.

A total of 155 patients who met the study inclusion criteria and were diagnosed with sporotrichosis at the reference center between June 2020 and June 2025 were analyzed. Of these, 52 (33.5%) were male and 103 (66.5%) were female (Table 1), with a mean age of 46.3 years (range, 6‒85 years). This inversion of the previously male-predominant epidemiological profile may reflect the social role of women as the primary caregivers of domestic animals, especially community and semi-domesticated cats, which constitute the main link in the zoonotic transmission chain^10^ ^,^ ^11^.

**TABLE 1:** Clinical-epidemiological data of patients with sporotrichosis treated between June 2020 to June 2025 at a reference center in Northern Brazil.

	Clinical-Epidemiological Data	Total	%
**Sex**	Male	52	33.5
	Female	103	66.5
**Age group**	6‒17 years	18	11.6
	18‒59 years	120	77.4
	≥60 years	17	11
**Origin**	Belém	149	96.1
	Ananindeua	5	3.3
	Santo Antônio do Tauá	1	0.6
**Feline exposure**	Prior contact	148	95.5
	Scratch, bite or contact with secretions	129	87.1
	Sick feline (skin lesion)	131	88,6
	Treatment	79	53.4
	Euthanasia or death due to the disease	34	23
Clinical Form*	Localized cutaneous	79	51
	Lymphocutaneous	73	47.1
	Severe/disseminated	2	1.2
	Ocular	3	1.9
Site of injury*	Upper limbs	120	77.4
	Lower limbs	21	13.5
	Face	11	7
	Trunk	10	6.4
**Medication**	Itraconazole	150	96.8
	Terbinafine	3	2
	Potassium Iodide	2	1.2
Healing time**	≤ 3 months	56	60.2
	4-6 months	32	34.4
	≥ 7 months	5	5.4

* Totals may exceed 100% because of multiple findings/forms per patient. ** 62 patients with loss to follow-up or still undergoing treatment/disease monitoring.

The concentration of cases in the capital city of Belém (Table 1) reinforces the urban nature of the disease and its relevance as an emerging public health problem in the Amazon, highlighting the need for integrated strategies for epidemiological surveillance and feline population control. 

Regarding epidemiological factors, ten patients reported prior contact with soil or decomposing organic matter, and only three reported exclusive exposure through this route, while the remaining patients also reported prior contact with cats. A total of 129 patients reported probable direct inoculation through cat scratches or bites (Table 1).

Of the felines evaluated, 131 (88.6 %) presented with skin lesions consistent with sporotrichosis, and 122 had a diagnosis confirmed by a veterinarian. Information regarding feline treatment was available for 113 cases, of which 16 were euthanized owing to clinical severity, 79 received treatment with itraconazole, and 18 died without veterinary intervention.

This study demonstrated a predominance of localized cutaneous (51.0%) and lymphocutaneous (47.1%) forms (Table 1), consistent with the most frequently reported clinical manifestations in the literature^12^. However, these findings differ from those reported by Cabeza et al. (2022), who identified the lymphocutaneous form as the predominant clinical presentation. Of the three patients with ocular involvement, two initially presented with the lymphocutaneous form, followed by the subsequent development of ocular lesions, whereas the third case was attributed to direct ocular inoculation. Consequently, the clinical forms listed in Table 1 are not mutually exclusive, as certain patients exhibited concurrent or progressive manifestations. The primary site of lesions was the upper limbs (77.4%), which is likely associated with the higher frequency of inoculation through cat scratches and bites affecting the hands and arms of the caregiver^13^.

Isolation and identification of *Sporothrix* species in culture are considered the gold standard for the diagnosis of sporotrichosis. Although direct mycological examination can be useful, it has low sensitivity and specificity in human sporotrichosis^3^. In this study, cultures were positive in 48.9% of cases. Clinical samples were collected and transported in sterile saline, with a processing time ranging from 24 to 48 hours between sample collection and culture inoculation. Fungal cultures were performed exclusively using Sabouraud Dextrose Agar and monitored daily for up to 30 days. The observed culture sensitivity (48.9%) was likely influenced by the intrinsically low fungal burden of chronic lesions, previous exposure to antibiotics or antifungal agents before clinical evaluation, and the time interval between sample collection and laboratory processing.

Histopathological examination of 87 cases demonstrated substantial complementary diagnostic value, primarily owing to the identification of asteroid bodies and granulomatous inflammatory patterns (Figure 1). The association between culture and histopathology increases the likelihood of diagnostic confirmation.

**FIGURE 1:**
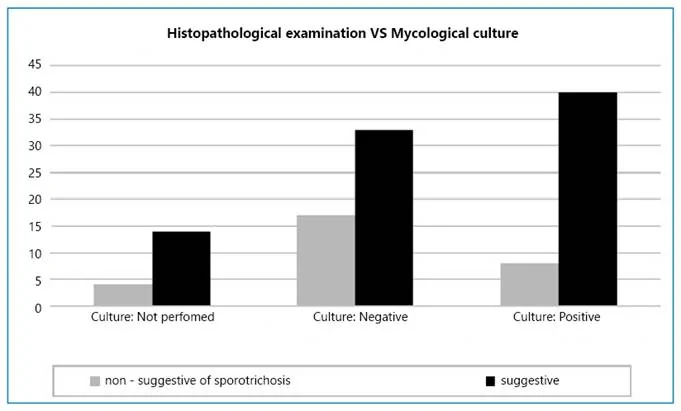
Relationship between histopathological findings and mycological culture results.

Itraconazole is the first-line drug of choice for the treatment of sporotrichosis, with well-established efficacy and a favorable safety profile, consistent with national recommendations^3^ ^,^ ^8^. In this series, 96.8% of patients were treated with itraconazole, with only three cases of treatment modification due to mild adverse events (namely, myalgia and gastrointestinal intolerance). 

Fifty-five patients (36.6%) received an initial itraconazole dose of 100 mg/day; among these patients, 10 (7.3%) required dose escalation to 200 mg, whereas one patient required an increase to 400 mg/day. A starting dose of 200 mg was administered to 94 patients (62.6%), of whom two required dose escalation to 400 mg. In one patient, a dose of 400 mg/day was prescribed from the beginning of treatment and maintained throughout the therapeutic course owing to the severity and extent of the disease. Potassium iodide (1‒2 g/day in children and 2‒4 g/day in adults) or terbinafine (250 mg/day), administered in 3.2% of cases, were reserved for pediatric patients and individuals with intolerance to itraconazole.

The mean treatment duration was 4.5 months, which is consistent with the 3- to 6-month treatment period reported in previous studies^14^ ^,^ ^15^. Regarding treatment outcomes, 93 patients achieved complete clinical resolution, 46 were lost to follow-up, and 16 remained under monitoring at the time of analysis. This high rate of loss to follow-up represents an important public health challenge, as premature treatment discontinuation may increase the risk of chronic and refractory lesions. All patients who achieved complete clinical resolution were discharged from outpatient care and underwent subsequent clinical follow-up, with no documented recurrences or therapeutic failures.

Nevertheless, effective control of the endemic sporotrichosis requires that human treatment be accompanied by integrated veterinary interventions, including the treatment of infected felines, health education, and the appropriate disposal of deceased animals to reduce environmental contamination and interrupt the zoonotic transmission cycle^6^.

In summary, the data presented in this study provide relevant insights for countries where sporotrichosis is endemic, emphasizing the strong clinical-epidemiological association between the disease and prior contact with cats, which was reported in most cases. These findings reinforce the importance of a thorough clinical history and public health education in preventing disease transmission. They also highlight the need to maintain a high level of diagnostic suspicion in endemic areas. Furthermore, mandatory and standardized reporting of sporotrichosis is essential for strengthening epidemiological surveillance and supporting the development of more effective regional policies aimed at reducing the incidence of this emerging zoonosis in the Amazon.

## Data Availability

Data-available-upon-request (medical records and personal information).
